# Survival from Cervical Necrotizing Fasciitis

**DOI:** 10.5811/westjem.2014.12.21553

**Published:** 2015-01-12

**Authors:** Jeniffer S. Gausepohl, Jonathan G. Wagner

**Affiliations:** Los Angeles County + University of Southern California Health Network, Department of Emergency Medicine, Los Angeles, California

## Abstract

Cervical necrotizing fasciitis (CNF) is an uncommon, yet clinically significant infection that rapidly progresses to involve the deep neck spaces. Early recognition and aggressive surgical intervention and debridement are important, as this disease is associated with a high morbidity and mortality. In this report, we present a case of CNF and descending mediastinitis from a non-odontogenic source in a patient presenting with neck swelling and odynophagia.

## CASE REPORT

**Figure 1:**
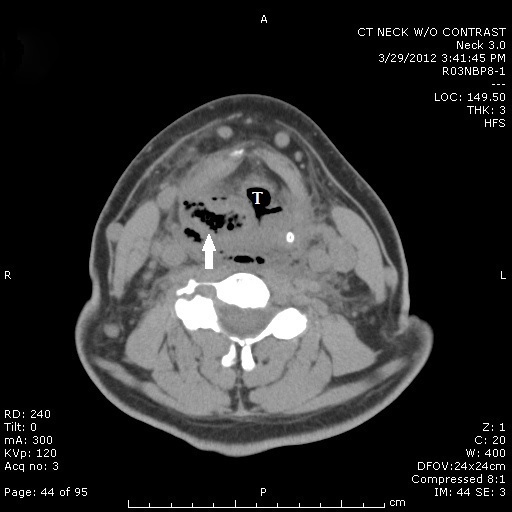
Computed tomography (CT) of the neck demonstrating soft tissue swelling, liquid and air collection within the oropharynx (arrow) near the trachea (labeled T).

**Figure 2:**
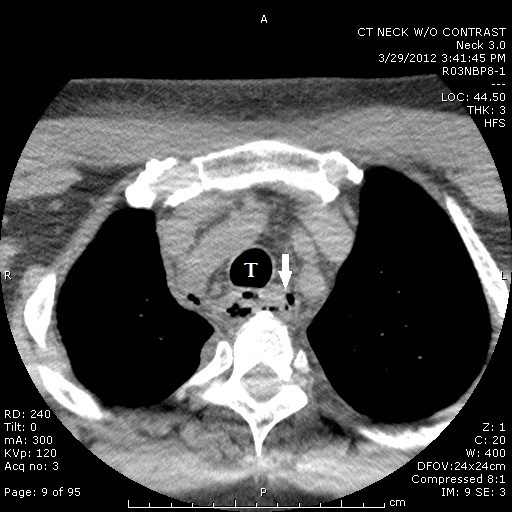
Computed tomography (CT) of the neck showing extensive edema and air within the upper mediastinum (arrow) tracking adjacent to trachea (labeled T).

A 59-year-old Caucasian man with past medical history significant for hypertension and hyperlipidemia presented to the emergency department with a four-day history of increasing throat pain and bilateral neck swelling. Associated symptoms included voice hoarseness, shortness of breath, dysphagia, and odynophagia. He denied recent dental procedures, upper respiratory tract infection or history of similar symptoms. Patient’s social history was negative for smoking, alcohol and illicit drug use. Initial vital signs were Blood Pressure 109/68, Pulse 101, Repiratory Rate 16, Temperature 98.1, and O_2_ saturation of 100% on room air. On exam, the patient was noted to have edema and tenderness to palpation of his entire right neck, with erythema tracking from the superior border of his right clavicle to the angle of the jaw on the right. No induration, crepitus or bullae were noted on the skin. Oral examination was remarkable for edema and erythema of the right anterior tonsil, without exudates or fluctuance, with mild deviation of the uvula to left. The laboratory values were significant for elevated leukocyte count of 29,000 with 89.9 % neutrophils and blood urea nitrogen and creatinine of 37 and 2.32, respectively. Computed tomography (CT) of the neck without contrast showed extensive edema in the oropharynx/hypopharynx, with edema and air within the retropharyngeal and danger space, as well as debris within the piriform sinus (Figures 1 and 2). Given the clinical exam, CT findings highly suspicious for “a gas-forming organism or necrotizing fasciitis,” and his laboratory results, blood cultures were drawn and intravenous clindamycin, vancomycin and ceftriaxone were empirically started. Otolaryngology was emergently consulted and the decision was made to immediately take the patient to the operating room for incision, drainage and washout.

Following nasotracheal intubation in the operating room (OR), the right neck was explored laterally into the retropharyngeal space. Purulent drainage was found to track via an overlying necrotic fascial plane into the parapharyngeal spaces as well as inferiorly into the superior mediastinum. Intraoperative gram stain showed gram positive cocci in both chains and clusters, gram negative rods and gram positive rods. The antibiotic regimen was changed to piperacillin/tazobactam and metronidazole (with discontinuation of clindamycin and ceftriaxone). Final wound culture grew *Streptococcus anginosus* and coagulase negative staphylococcus. The patient remained intubated postoperatively, and a repeat CT was performed on postoperative day 4 due to persistent leukocytosis. A residual phlegmon in the bilateral piriform sinuses was discovered, and the patient was then taken back to the OR for repeat right neck exploration, direct laryngoscopy, and bilateral incision and drainage of the peritonsillar space. Intra-operatively, cardiothoracic surgery was consulted to perform an open lateral thoracotomy to drain a posterior mediastinal phlegmon.

Despite repeat drainage, the patient began to decompensate, requiring multiple vasopressors to maintain adequate perfusion, and he suffered from persistent fevers, acute renal failure and transaminitis (aspartate aminotransferase 4202, alanine aminotransferase 1922). His leukocytosis continued to rise, peaking at 51,600. On postoperative day 7, the patient suffered a cardiac arrest requiring one round of chest compressions and epinephrine before return of spontaneous circulation. On postoperative day 9, his multisystem organ failure began to improve and the patient was slowly weaned off vasopressors, with successful extubation on postoperative day 12. He was discharged to a rehabilitation institution on postoperative day 21 with a peripherally inserted central catheter line to continue vancomycin, clindamycin and metronidazole for a total of four weeks. On recent follow-up with otolaryngology six weeks after his discharge, he was noted to be doing well and has elected to undergo cosmetic revision of right neck scar, the date of which is to be determined.

## DISCUSSION

Cervical necrotizing fasciitis (CNF) is a rare polymicrobial infection of the fascial planes of the neck associated with high morbidity and mortality. With isolated CNF, mortality approaches 20%, and when associated with extension into the mediastinum and sepsis, rates as high as 41% and 64% have been reported respectively.^1^ Predisposing factors associated with development of CNF include diabetes mellitus, poor dental hygiene, obesity, alcoholism, and immunocompromised states.^2^,^3^ CNF is a destructive and rapidly advancing form of necrotizing fasciitis that most commonly originates from odontogenic or pharyngeal sources, with the signature characteristic of this disease being necrosis of the layers of the fascia underlying the skin and surrounding vasculature.^4^,^5^ Due to a plentiful blood supply, necrotizing fasciitis of the head and neck is rare in comparison to other regions of the body, such as the limbs and perineum.^1^ Early death in CNF is often a result of airway compromise, while later mortality is related to sepsis and septic shock. The gas-forming organisms associated with this disease can separate fascial planes and quickly travel to the thorax and mediastinum via the “danger space,” an anatomical pathway that connects the base of the skull to the diaphragm. Factors associated with development of mediastinitis include: infection of the pharynx, presence of gas, and use of glucocorticoids prior to hospital admission.^7^ Mediastinitis has a high rate of mortality, and along with septic shock, is the most dismal prognostic indicator in CNF.^1^,^7^

Necrotizing fasciitis is well known for its rapid tissue spread and destruction, while having a benign external appearance. Skin evidence of the disease includes grey-patchy discoloration, bullae, or frank necrosis. Despite these characteristic external findings, they typically occur late in the course of the disease. Early signs that should raise awareness for the possibility of necrotizing fasciitis include pain out of proportion to touch or anesthesia/hypoesthesia to the affected area.^5^ Diagnosing CNF is largely based on clinical presentation, with findings on CT, such as fat stranding and gas tracking along fascial planes, serving to increase the probability of the diagnosis. The final diagnosis, however, is confirmed by surgical exploration.^6^ In the majority of cases, necrotizing fasciitis is a polymicrobial synergistic infection with both aerobic and obligate anaerobic bacteria (streptococci and enterobacteriaceae the most commonly implicated species).^8^,^9^ Treatment includes early incision and drainage, aggressive debridement, broad spectrum intravenous antibiotics, airway management, and close monitoring in an intensive care unit.^1^ As with all types of necrotizing fasciitis, drainage and surgical exploration is the most important part of treatment. Early operative intervention has been shown to improve outcome in CNF, and conversely, a delay in debridement of more than 24 hours is correlated with worsening mortality.^8^
